# Entrepreneurship in the twenty-first century

**DOI:** 10.1007/s11187-021-00542-0

**Published:** 2021-10-05

**Authors:** John Haltiwanger

**Affiliations:** 1 University of Maryland, College Park, USA; 2 NBER, Cambridge, USA

**Keywords:** Entrepreneurship, Job creation, Productivity growth, L25, L26, O33

## Abstract

Employer business startups contribute disproportionately to job creation, innovation, and productivity growth. This contribution is dynamic and complex involving much trial and error. Most startups fail or do not grow but a small fraction grow rapidly contributing substantially to economic performance. In the USA, there has been a decline in startup rate and the share of activity accounted for by young firms over the last couple of decades. This decline has accelerated and become pervasive in the post-2000 period even in innovative-intensive sectors. The flip side of this change is an accompanying increase in the share of activity accounted for by mega (10,000 +) firms in the post-2000 period. While both benign and adverse factors may underlie these structural changes, the post-2000 period has also exhibited a decline in productivity growth along with indicators of business dynamism. The global pandemic has had a major adverse impact on health, morbidity, daily life, and economic activity. However, there has been a surge in new business applications that may signal a turning point in entrepreneurial activity.

## Abstract (plain language summary)

In the twenty-first century, we have witnessed a sharp decline in the number of new startups in the USA across all sectors, including those sectors considered high-tech and innovative. The reason why this has occurred is still open for discussion and debate. This development is worrying as new startups contribute disproportionately to job creation, innovation, and productivity growth. Alongside this, the global pandemic has brought about a massive reduction in economic activity and its restructuring. At the same time, there has been a distinct increase in applications for new businesses, especially in industries that facilitate remote interactions between businesses and consumers and businesses and workers. It is an open question whether this reverses the long-run trend of declining entrepreneurship that the USA has been experiencing or if this has only brought about a temporary change. There will be plenty of opportunities for future research to disentangle the reasons for and persistence in the business dynamics seen in US data.

Much evidence shows that new employer startups contribute disproportionately to job creation, innovation, and productivity growth. Entrepreneurs both are induced by and induce innovation. This contribution of new employer businesses reflects patterns of trial and error and experimentation. Most new businesses fail within the first five years of entry and, conditional on survival, most do not grow (see Haltiwanger et al., 2013, and Decker et al., 2014). However, a relatively small fraction of young businesses grow very rapidly and it is these high growth firms that are especially important for job creation that persists, innovation and productivity growth.^1^

While high growth firms are more likely to be found in innovative-intensive sectors (see, e.g., Acemoglu et al., 2018, Guzman and Stern, 2020, and Decker et al., 2016), young businesses in these sectors exhibit enormous dispersion and skewness in post-entry growth and other performance measures.^2^ Indeed, consistent with the theories of experimentation and learning from Gort and Klepper (1982) and Jovanovic (1982), sectors that are undergoing rapid restructuring and innovation have a distinct dynamic pattern of entry, productivity dispersion, and productivity growth (see Foster et al., 2021). Specifically, surges of entry in innovative sectors first yield an increase in dispersion of productivity and growth rates across firms in the sector along with a decline in productivity growth. It is only after an experimentation and learning period including a shakeout process that productivity growth ensues.

Evidence shows that this characterization of the contribution of entrepreneurship to innovation is consistent with evidence from virtually the entire twentieth Century. However, in the twenty-first century, there has been a notable decline in the contribution of young businesses in the USA even in innovative-intensive sectors such as high tech. Much ongoing research attempts to explain this decline in entrepreneurial activity (see, e.g., Davis and Haltiwanger, 2014, Decker et al., 2014, 2016, Decker et al., 2020, Karahan et al., 2021, Salgado, 2017) and the accompanying increasing dominance of large, multi-national firms (see, e.g., Autor et al., 2020).

The global pandemic starting in early 2020 and continuing into 2021 has generated a massive downturn in economic activity that may be a turning point for entrepreneurship in the twenty-first century. The pandemic has had enormous adverse consequences for health, morbidity, social interaction, and economic activity. The negative effects of the pandemic are staggering. Perhaps in reaction to the large negative but uneven shocks to economic activity, there has been a surge in applications for new businesses in the pandemic that has been large and distinct (see Haltiwanger, 2021). The total number of applications in 2020 is the highest by far compared to all years for which the data have been available (since 2004). The increase from 2019 to 2020 in total applications exceeds 20% which is double the growth rate in any other year. The increase is in applications for both likely employers and likely non-employers. The increase in 2020 is wholly accounted for by a surge in applications in the second half of 2020. Based on historical patterns, this surge in applications should result in a surge in new employer and non-employer businesses.

The surge in new business applications has been uneven across sectors. Ten 3-digit industries out of about 100 3-digit industries account for 75% of the surge. Dominant industries include Non-store Retail (alone accounting for 33% of the surge); personal services; professional, scientific and technical services; administrative and support services; truck transportation; and accommodation and food services. Given that existing small businesses in Retail Trade and Accommodation and Food Services have suffered especially large declines in the pandemic, these patterns are consistent with restructuring induced by the pandemic (see Buffington et al., 2021).

As the economy recovers from the pandemic, an open question is the extent to which the changes in business operations (especially increases in remote/telework activity) observed during the pandemic will persist. The extent to which these changes “stick” is likely to vary across types of businesses and locations. The shift towards e-commerce is likely to stick as this reflects a pre-pandemic trend. Viewed from this perspective, the pandemic may be accelerating ongoing trends and the surge in business applications is part of this process.

This paper explores the latest evidence on these changing patterns of entrepreneurship in the twenty-first century using the Business Dynamic Statistics (BDS) and the Business Formation Statistics (BFS) for the USA. Section 1 describes the data. Section 2 reviews the basic facts on the evidence of declining entrepreneurship in the USA using sectoral variation to help shed light on the alternative hypotheses that have been proposed. Section 3 reviews the evidence on new business applications from the BFS. The BFS offers high frequency (weekly and monthly) real-time evidence on new business applications. Sectoral variation again provides key insights to interpreting the surprising surge in new business applications during the pandemic. Concluding remarks are provided in Section 4.

## Data (BDS and BFS)

The analysis of young-firm activity in this paper relies heavily on two US Census Bureau statistical products: BDS and BFS.^3^ The BDS includes employment statistics by firm size, firm age, and industry tabulated from micro data in the Longitudinal Business Database (LBD).^4^ The LBD covers the universe of firms and establishments in the non-farm business sector with at least one paid employee. Employee counts pertain to the payroll period covering the 12th of March in each year from 1976 to 2018. Firm characteristics reflect the national firm. Firm age in the BDS reflects the age of its oldest establishment when the firm first became a legal entity. For a startup business comprised all new establishments, firm age is initially set to zero. For firms newly created from one or more existing establishments through a merger, spinoff, or corporate reorganization, firm age is initially set to the age of its oldest establishment. From that point forward, the firm ages naturally as long as it exists. Simple ownership changes do not trigger a change in firm age, and the BDS concept of business startups reflects new firms with only age-zero establishments. These features of the BDS are a major strength, as they ensure that our young-firm activity measures and their evolution are not distorted by firm restructurings and ownership changes.

For simplicity and brevity, the analysis in this paper focuses on two age groups: “young” firms that are ten years or less, and “mature” firms that are at least eleven years old.^5^ Using these definitions, the BDS enables us to track young- and mature-firm activity measures at the national and detailed (4-digit NAICS level) from 1987 to 2018. Firm size is based on the number of workers at the national firm. In this paper, firm size is based on the size of the firm in *t*-1 (except for startups which use size in *t*). Small firms are defined as firms with less than 500 employees, large firms are firms with 500 or more employees, and mega firms are those with 10,000 or more employees. The BDS enables classifying firm size by detailed industry and firm size by firm age but not a three-way classification.

The BFS is derived from administrative data from the Internal Revenue Service (IRS) on Employer Identification Number (EIN) applications. All employer businesses in the USA are required to have an EIN to file payroll taxes. New non-employer businesses also file for an EIN if forming a partnership or an incorporated business. Even new sole proprietor non-employers often file for an EIN to facilitate their business activity (e.g., working with other businesses or opening a business bank account). The EIN application form includes the name and address of the applicant and business, business start date, type of business entity, principal industry, and planned date of initial wage payments (if applicable). The filing date and business location information are used to aggregate individual applications to weekly and monthly frequency. The IRS transmits these applications to Census on a weekly flow basis in virtually real time.

The detailed information on the application permits decomposing new business applications (BA) into likely employers and likely non-employers. Businesses that have a high propensity of becoming an employer business based upon, for example, the application indicating planned wages are designated as High-Propensity Business Applications (HBA). Consistent with Bayard et al. (2018), evidence presented in this paper shows that there is a tight relationship between HBA and actual new employer startups over the subsequent 8 quarters. The difference between BA and HBA is referred to as likely non-employers (NHBA) in this paper, and the analysis in Haltiwanger (2021) shows that fluctuations in NHBA closely track fluctuations in non-employers. The public domain BFS also includes series by geography (state) and industry. The geographic and industry variation permits analysis of the dispersion in entrepreneurial activity across sectors and locations.

## The decline of young businesses and the rise of “superstar” firms

### Basic facts

**Fig. 1:**
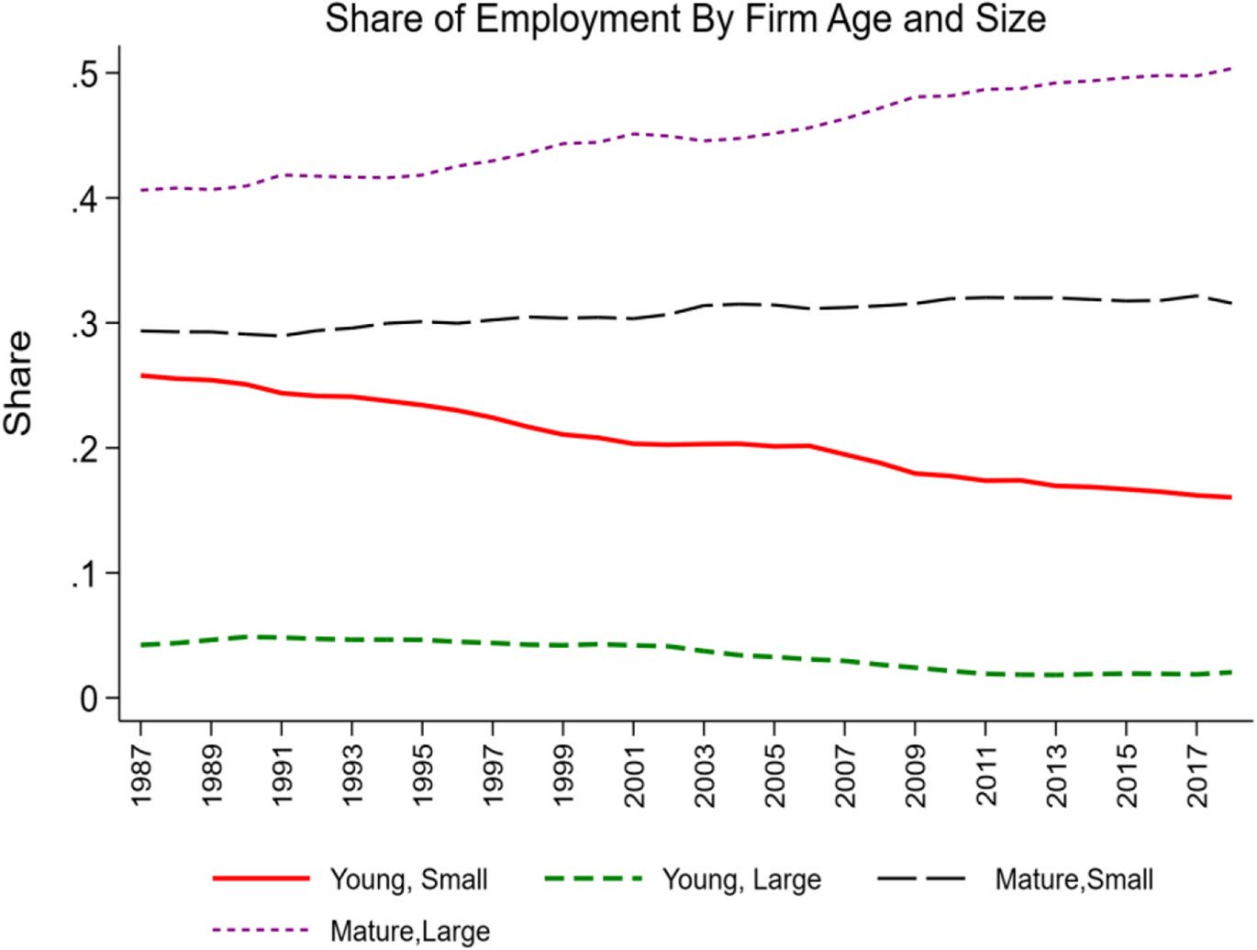
Changing composition of employment by firm age and firm size. Source: Business Dynamics Statistics. Note: young < 11, mature 11 + , small < 500, large 500 +

The shift away from young, small businesses towards large, mature businesses is depicted in Fig.1. At an economy-wide level, this shift has been ongoing since the late 1980s. Figure 1 masks substantial heterogeneity across industries in the changing structure of businesses by firm age and firm size. The BDS does not provide information by three-way characteristics (i.e., it does not provide firm age by firm size by industry) so the analysis here is for two sets of two-way pairs (by firm age and industry and then by firm size and industry).

**Fig. 2:**
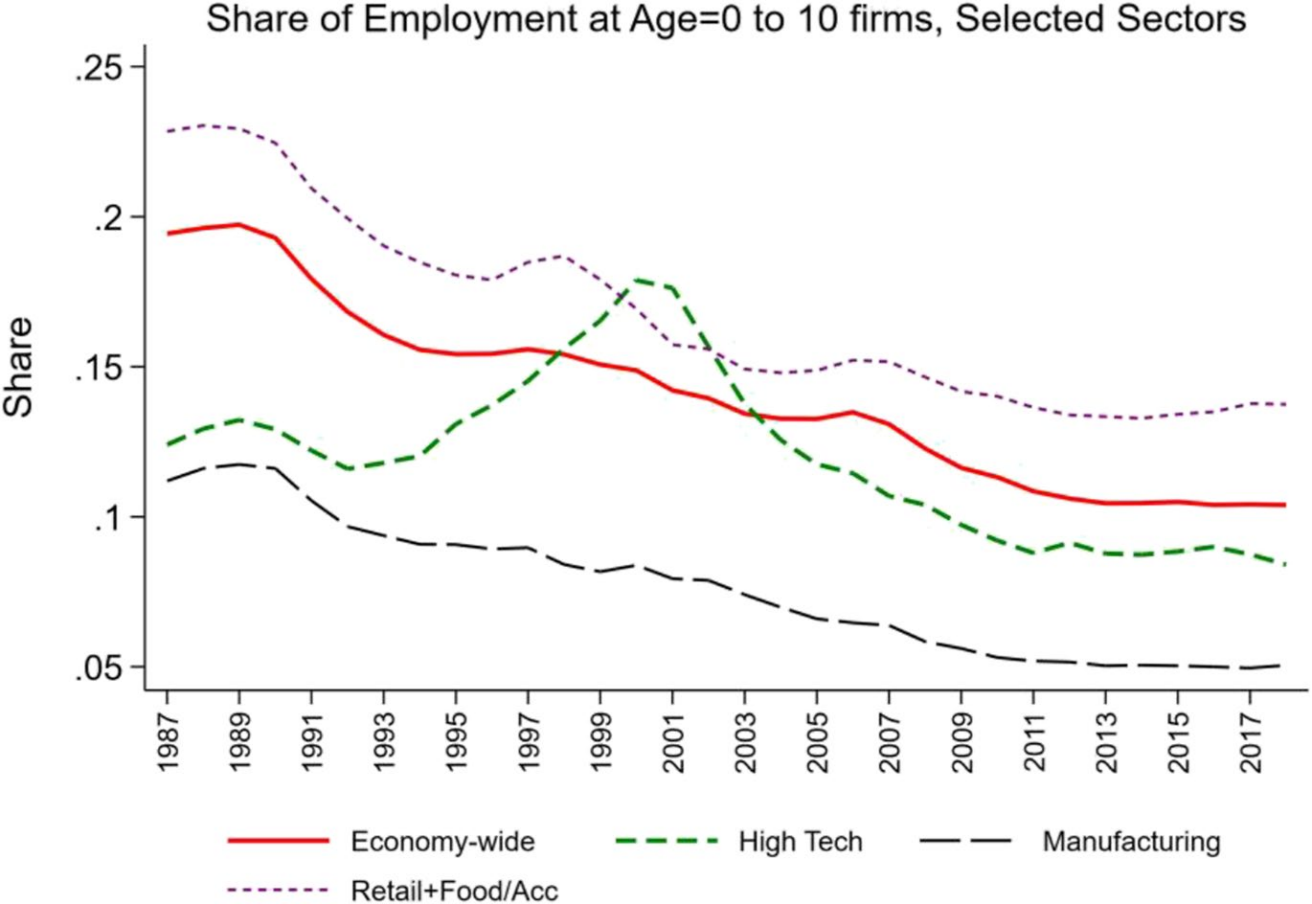
Changing share of employment at young firms, selected sectors. Source: Business Dynamics Statistics

**Fig. 3:**
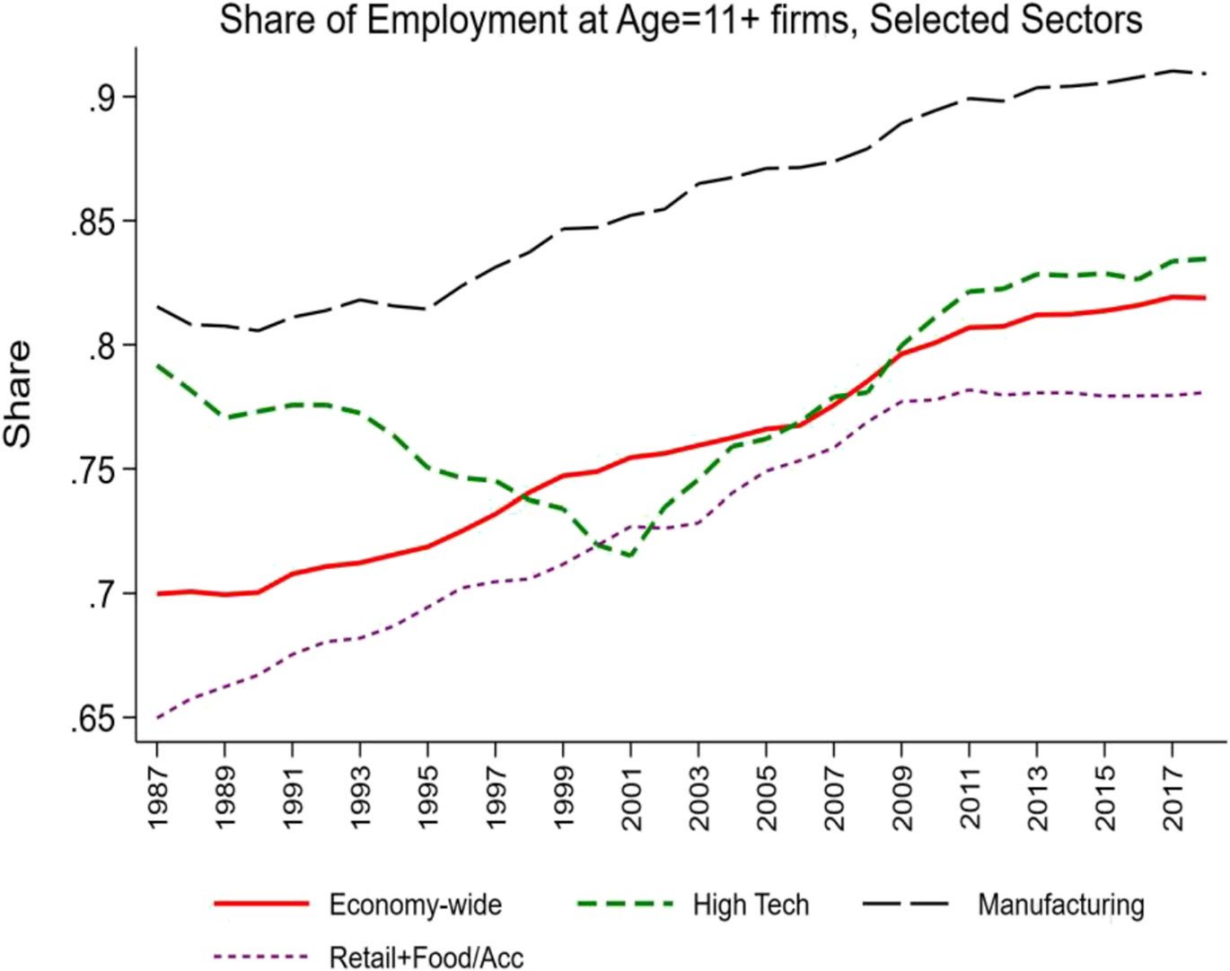
Changing share of employment at mature firms, selected sectors. Source: Business Dynamics Statistics

Figures 2 and 3 show the evolving share of employment at young and mature firms by selected sectors. The 4-digit NAICS BDS statistics are used to classify industries into high-tech industries following Decker et al. (2020). This classification is based on the science, technology, engineering, and mathematics (STEM) intensity of the industry in terms of occupation mix and includes all of Information and Communication Technology (ICT) industries plus industries in biotech (e.g., high tech includes NAICS 5417, Scientific and Research Services which in turn includes Research and Development in Biotechnology, NAICS 541711).

Industries such as manufacturing and Retail Trade (and Food and Accommodation Services)^6^ have exhibited long-run declines in the share of employment accounted for by young firms and the accompanying rise in the share of employment activity accounted for by mature firms. In contrast, the high-tech sector exhibited an increase in the share of activity accounted for by young firms through the early 2000s, but it has declined substantially since then.

**Fig. 4:**
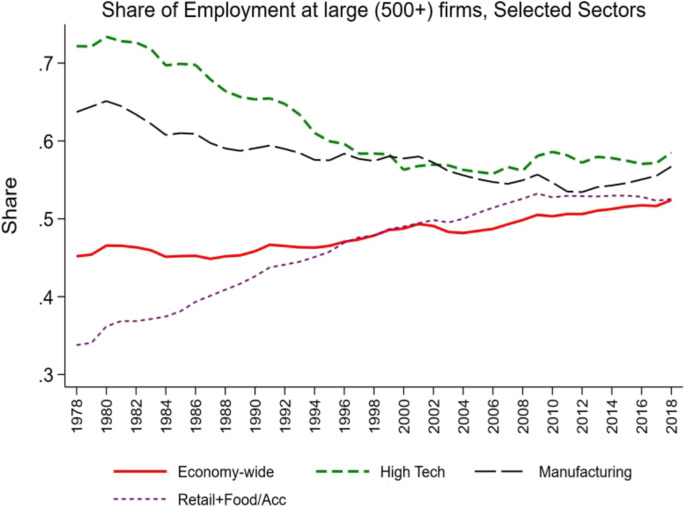
Changing share of employment at large firms. Source: Business Dynamics Statistics

**Fig. 5:**
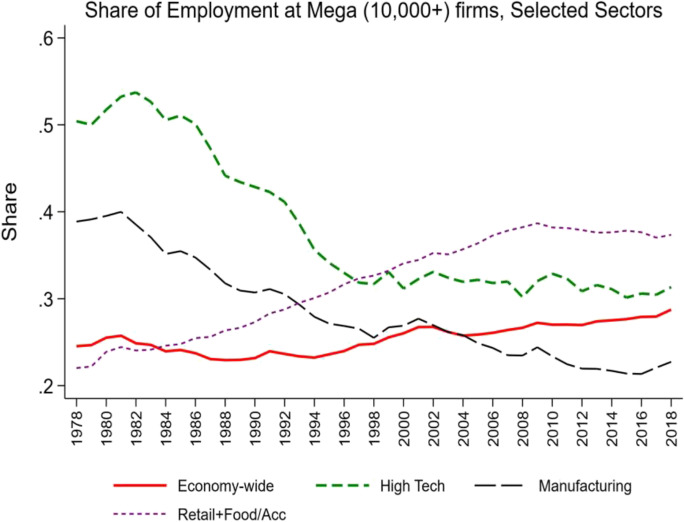
Changing share of employment at mega firms. Source: Business Dynamics Statistics

There has been a growing shift in employment to large and even mega firms over this period (see Figs.4 and 5). These patterns have also been uneven across sectors and industries. Retail Trade (and Food and Accommodation Services) has exhibited the largest and steadiest increases. Manufacturing has exhibited a long-run secular decline which is striking given the shift towards more mature manufacturing firms. The high-tech sector has exhibited a decline in the share of employment at large and mega firms through 2000, and it has been relatively flat since then.

**Fig. 6:**
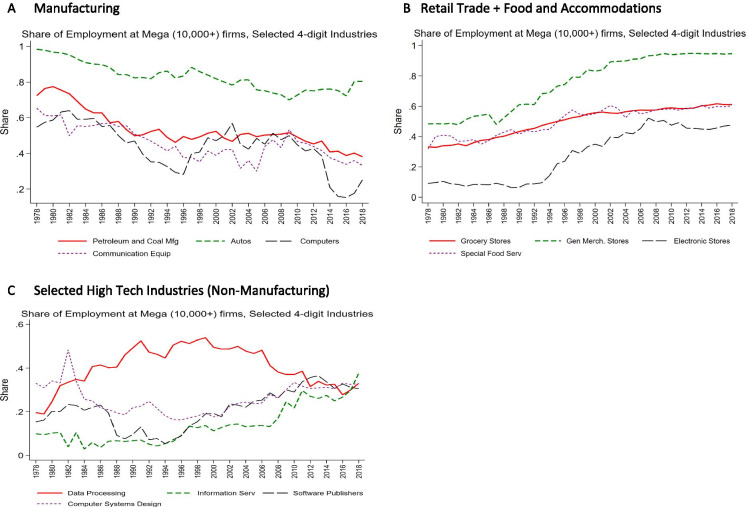
Changing share of employment at mega firms, selected detailed industries. **A** Manufacturing. **B** Retail Trade + Food and Accommodations. **C** Selected high-tech industries (non-manufacturing). Source: Business Dynamics Statistics

Figure 6 shows that some of these patterns reflect distinct differences at a more detailed industry level. For manufacturing, there has been a secular decline in the share of employment at mega firms both for traditional manufacturing such as autos and petroleum and coal products and for high-tech industries such as communications equipment and computer manufacturing. For Retail Trade, there has been especially large increases in the share of employment at mega firms in detailed industries such as (other) general merchandise stores. For the non-manufacturing components of high tech (e.g., software publishing), there has been an increase in the share of employment at mega firms in the post-2000 period.

Over this period, there has been a shift within high tech away from manufacturing to the non-manufacturing components. Figure 6 helps account for the overall decline in mega firms in high tech given this shift as the manufacturing components have higher mega shares than the non-manufacturing components. Still, it is interesting that key non-manufacturing components of high tech have rising mega shares especially post-2000.

### Interpretation

The economy-wide trend decline in activity accounted for by young businesses has led many to seek mono-causal explanations for the decline. These include the aging of the population (see, e.g., Karahan et al., 2021, and Hopenhayn et al., 2020) and skill-biased technical change that favors larger, mature firms (Salgado, 2017). While these common factors are likely at work, the distinct patterns at the sectoral and industry level raise questions about mono-causal explanations of the changing structure of activity. Retail Trade has been undergoing a structural transformation for decades away from single-unit establishment firms (“mom and pop firms”) to large, national chains (see, e.g., Foster et al., 2006, 2015). Globalization and advances in information technology facilitate more efficient distribution networks for large, global firms particularly in sectors such as Retail Trade. The evidence shows that establishments of large, global firms are substantially more productive than single-unit establishment firms within the same sector. As such, this shift towards mega firms in Retail Trade has been productivity-enhancing. This discussion suggests that the rise of superstar firms in Retail Trade is thus potentially associated with benign factors.

It is difficult to make the case that benign factors account for all the patterns in the data. The innovative-intensive industries in high tech exhibited a surge in young firm activity in the 1990s and only exhibit a decline in the post-2000 period. Foster et al. (2021) show a tight connection between a surge in entry and subsequent productivity growth in the high-tech industries over the 1990s and beyond. Using detailed 4-digit NAICS sectors and 3-year non-overlapping periods, they find that a surge in entry in one 3-year period first leads to a rise in within-industry dispersion in productivity and actually a mild decline in industry-level productivity growth in the next 3-year period. However, in the subsequent 3-year period, there is a surge in industry-level productivity growth and a decline in within-industry productivity dispersion.

These patterns for high tech are consistent with the models and evidence in Gort and Klepper (1982) and Jovanovic (1982) that highlight that innovation both is induced by and induces entrepreneurship. Gort and Klepper (1982) provide examples over the entire twentieth century showing that industries undergoing high pace of innovation first observe a surge in entry, then a shakeout process and then the growth of the surviving firms.

**Fig. 7:**
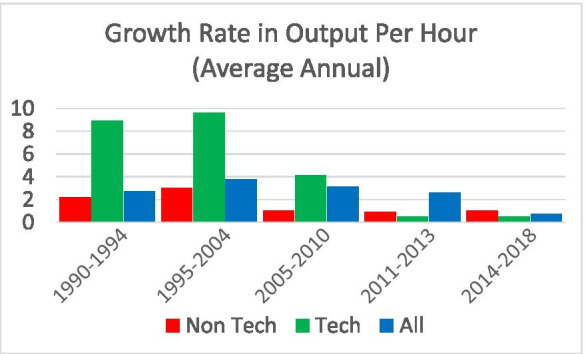
Declining productivity growth post-mid 2000s. Source: Bureau of Labor Statistics

Viewed from this perspective, innovation and entrepreneurship go hand in hand especially in industries where experimentation and trial and error are a critical part of the innovation dynamics. Figures 2 and 3 can be interpreted as reflecting a period where one key part of the economy (the industries in the high-tech sector) underwent these type of dynamics. Figure 7 provides supportive evidence consistent with Foster et al. (2021) showing that the surge in productivity in the 1990s and 2000s was driven by the high-tech component of the economy. Since the mid-2000s, Figs. 2, 3, and 4 together indicate that there is little evidence of any sector seeing the type of surge in young firm activity and the accompanying surge in productivity growth that the high-tech sector exhibited in the 1990s and early 2000s. It is also striking that in this era of rising mega firms in the high-tech sector (Fig. 6), productivity growth has slowed in the high-tech sector.

**Fig. 8:**
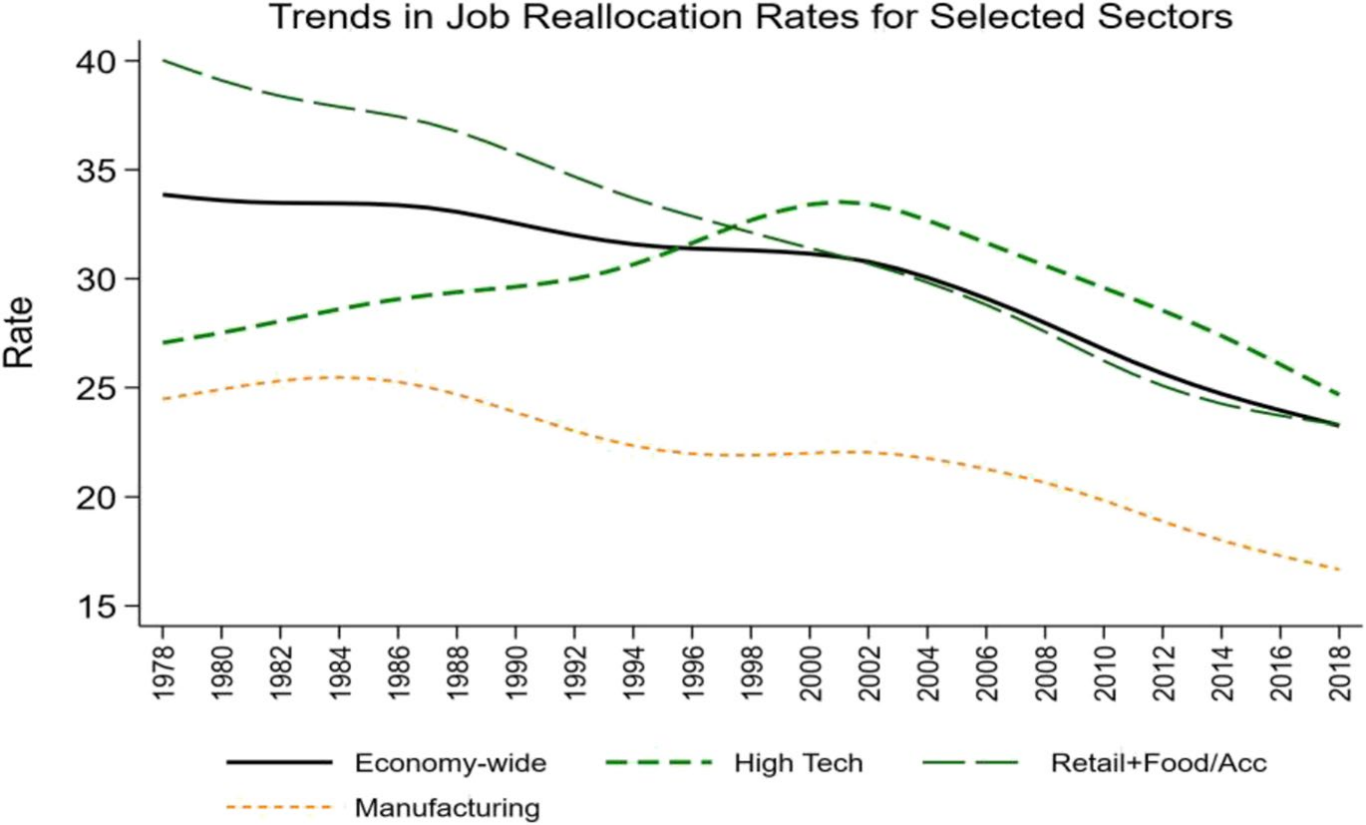
Declining dynamism. Source: Business Dynamics Statistics

An open question is why there has been this coincident slowdown in productivity growth and a pervasive decline in young firm activity in the post-2000 period. One hypothesis is that innovation itself has slowed down (see, e.g., Gordon, 2015). Alternatively, but perhaps not unrelatedly, there have been other structural changes in the economy over this period. Accompanying the decline in young share activity is a decline in indicators of business dynamism (see Fig. 8). Figure 8 shows considerable heterogeneity in the patterns of the decline in business dynamism across sectors. These differences largely mimic the changes in the share of young firm activity in Fig. 2. This is not surprising since young businesses exhibit a higher pace of business dynamism. However, Decker et al. (2014, 2020) show that the changing age composition of firms can account for at most 30% of the decline in business dynamism.

The causes of this decline are not yet well understood but Decker et al. (2020) provide evidence of an increase in adjustment frictions especially amongst young firms. Specifically, they find that the responsiveness of employment growth and investment to realizations of productivity have declined in the post-2000 period. They also find an increase in dispersion of revenue productivity measures within industries consistent with rising frictions or distortions. In related work, DeLoecker et al. (2020) find evidence of rising average and dispersion in markups across firms.

It is possible the benign and adverse effects of the changing age and size structure of firms are related. The rise of superstar firms from globalization, information technology, and other factors such as increased importance of network externalities may also underlie the rising markups and declining dynamism. Large incumbents have an incentive to engage in more defensive innovations (see, e.g., Akcigit and Kerr, 2018) and to stifle competition by killer acquisitions (Cunningham et al., 2021) of young, innovative startups.

This perspective leads to a less optimistic outlook for future growth and the role of entrepreneurship in contributing to that growth in the twenty-first century. Not all share that less optimistic view given the major advancements in technology that seem to be just around the corner in terms of widespread adoption such as AI, robotics, and automation (see, Brynjolfsson and Mitchell, 2017). Indeed, Brynjolfsson et al. (2020) argue that the more recent advances have greater “J-curve” effects in terms of the disruption and slow diffusion process that often accompanies the introduction of innovation into the market place. While these arguments are interesting, the long history of young firms being critical in the experimentation with new technologies raises questions about whether the introduction of these new technologies will somehow be different in the twenty-first century.

## The surge in new business applications in the pandemic

### Basic facts

**Fig. 9:**
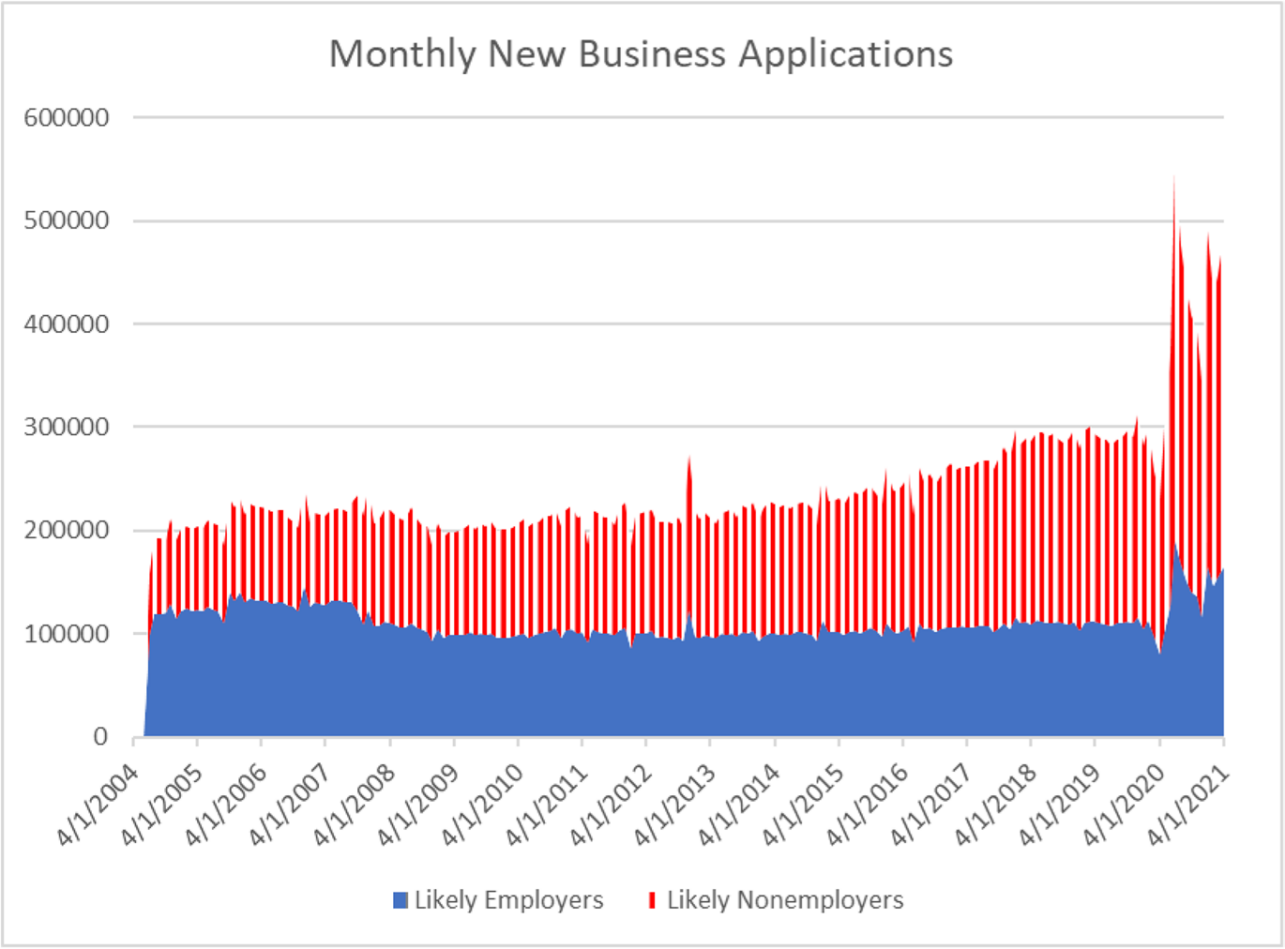
Applications for new businesses for likely employers and non-employers. Source: Business Formation Statistics

The patterns of new business applications (BA, HBA, and NHBA) from 2004:m7 through 2021:m4 are shown in Fig. 9. The upward trend in NHBA and the downward trend in HBA from 2004:m7 through 2020:m4 are evident. At the outset of the pandemic, there is a sharp decline in BA, HBA, and NHBA in March through May 2020. However, by June 2020, the number of applications of all types exceeds any month from January 2019 through February 2020. The surge in applications of all types peaks in July 2020 but the numbers in August 2020 to April 2021 exceed the number of applications in all prior months back to 2004:m7 for BA and NHBA and in all prior months back to 2007:12 for HBA. April 2021 is the third highest month on record for all series.

**Fig. 10:**
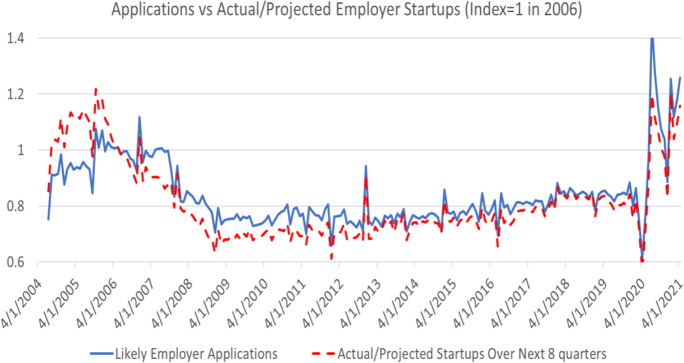
Tight relationship between HBA and new employer business startups. Source: Business Formation Statistics

HBA tracks actual and projected transitions to new employer startups as shown in Fig. 10. Indices are depicted in Fig. 10 given differences in levels, and the projected transition series is for transitions to employer business over the next 8 quarters. The transition rate is lower than 50% (see Bayard et al., 2018) even for HBA, highlighting that HBA should be interpreted as an indicator of nascent entrepreneurship in terms of potential new employer startups. However, since the correlation in the monthly index series is 0.88, it is apparent that variation in this indicator of nascent entrepreneur closely tracks variation in new employer startups. The BFS does not provide indicators of actual or projected new non-employers. However, a comparison of NHBA with published non-employer statistics indicates a tight relationship as discussed in Haltiwanger (2021).

**Fig. 11:**
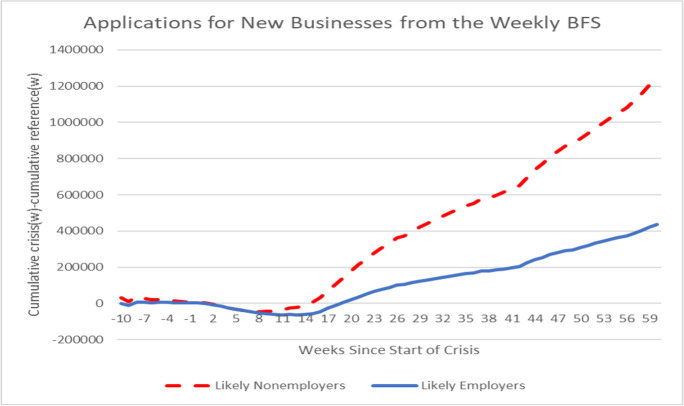
Surge in new business applications during COVID-19 crisis. Source: Business Formation Statistics. Note: week 0 in crisis is week ending March 13, 2020. Week 0 in reference period is week ending March 10, 2018

The weekly data enable a detailed comparison of the dynamics of new business applications in the COVID-19 Recession. A simple event study characterization of the dynamics is instructive following Dinlersoz et al. (2021) and Haltiwanger (2021). The reference week (0) is defined as week 10 of 2020 (the week ending March 13, 2020. The cumulative changes in applications (backwards and forwards) relative to week 10 of 2020 are first calculated. Then, the same computation is made for week 10 of 2018. Presented in Fig. 11 is the cumulative differences.

Prior to the crisis (i.e., prior to week 10 in 2020), applications for likely employers in the first 10 weeks of 2020 were similar to those for the base period of 2018. Shortly after mid-March 2020, applications fell initially relative to the similar period in 2018 but then have risen dramatically. By early May 2021, new applications for likely employers were almost 500 K greater over a similar period from March 2018 through early May 2018. For likely non-employers, the first part of 2020 had slightly higher applications than the comparable first part of 2018. Applications for non-employers also declined initially early in the crisis but then quickly rebounded. By early May 2021, new applications for likely non-employers were more than 1.2 million greater than a similar period from March 2018 through early May 2019.

**Fig. 12:**
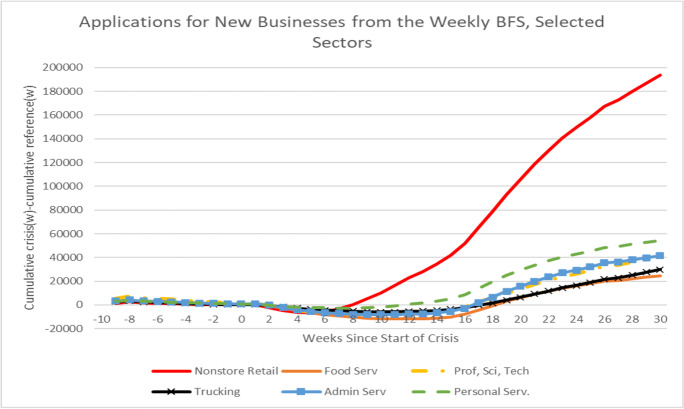
Surge in new business applications during COVID-19 crisis. Top contributing 3-digit industries. Source: Business Formation Statistics. Note: week 0 in crisis is week ending March 13, 2020. Week 0 in reference period is week ending March 9, 2019

The surge in new business applications in the pandemic has been uneven across industries. A special release in October 2020 of the weekly BFS covering 2019:w1–2020:w40 provides 3-digit industry detail for overall applications. Figure 12 presents the same type of exercise as in Fig. 11 for selected 3-digit NAICS sectors. Since these data are only available only starting in 2019:w1, the benchmark reference period is 2019 in Fig. 12 rather than 2018. The surge in new applications is greatest for Non-store Retailers which increased its number of applications by about 200,000 over this period. In 2019, Non-store Retailers accounted for only 9% of overall applications. Thirty-three percent of the increase in applications from 2019 to 2020 is accounted for by this industry alone. Other industries with large increases include Personal Services; Professional, Scientific, and Technical Services; Administrative Services; and Trucking and Food Services.

### Interpretation

As emphasized by Dinlersoz et al. (2021) and Haltiwanger (2021), this surge in new business applications in the pandemic differs greatly from the patterns in the Great Recession. In the Great Recession, applications for likely new employers declined sharply accompanied by a decline in actual new employer businesses. Explaining the reasons for these patterns is an active area for research. However, some factors already appear to be important. First, financial conditions are dramatically different across these episodes. The financial crisis that is at the root of the Great Recession included a decline in housing prices, a decline in net worth for households, and substantial challenges for bank balance sheets. Small business lending collapsed over this period. Some of this likely reflected demand side factors but Davis and Haltiwanger (2019) identify a credit supply channel that adversely impacted young businesses. In contrast, financial markets have been robust in the COVID-19 Recession with housing prices rising and financial intermediaries much healthier in this period.

Secondly, the COVID-19 Recession has induced a change in the structure of the US economy towards more remote activity and this provides incentives for new businesses to explore such potential opportunities. More broadly, the pandemic surge in business applications is associated with intensified restructuring on several dimensions. For one, entry by itself is an important component of restructuring in terms of business turnover. Second, the uneven patterns across sectors that change rapidly over a short period of time suggest that the restructuring has an important between-sector component. The sectors with especially high business application rates provide guidance about the nature of this restructuring. The dramatic rise in sectors such as Non-store Retail is consistent with the shift towards remote interactions between businesses and consumers.

Does this surge in new business applications imply there will be a surge in job creation, innovation, and productivity growth over the next few years? Related to the theme of this article, has the pandemic been a turning point in reversing the decline in entrepreneurship? Obviously, this is an open question but in considering these possibilities, several factors are important to consider. First, as discussed above, most new employer businesses fail within the first five years after entry and conditional on survival, most do not grow. A relatively small fraction of young businesses grow very rapidly and it is these high growth firms that are especially important for job creation that persists, innovation and productivity growth. All of this implies that it will take some time before we understand the implications of this surge in applications for likely new employers.

The surge in applications for likely non-employers is also of interest as an indicator of the changing structure of the economy. The number of non-employer businesses has been on an upward trend over the last 15 years or so with rapid acceleration in the post-2010 period. The surge in applications for likely non-employer thus also may reflect at least in part an acceleration of pre-pandemic trends. However, several factors suggest the surge is more complicated. For one, the surge in applications for likely non-employers are for those applying for EINs. The upward trend in non-employers pre-pandemic over the last decade has been dominated by the ridesharing industry (see, e.g., Abraham et al., 2021). Most of the non-employers in the ridesharing industry are sole proprietors that are not required to have an EIN. In contrast, the surge in applications for likely non-employers in the pandemic are in sectors that reflect promoting remote activity such as Non-store Retailers. Just as for new employer startups, an open question is whether new non-employers also will “stick.” A closely related question is whether the new non-employer business activity will mostly be stopgap or supplemental as in the past. Or alternatively, are we seeing an increase in the share of individuals where non-employer activity is the primary or only source of work activity?

## Concluding remarks

Whither entrepreneurship? In the USA, there are mixed signals. On the one hand, there is a secular decline in employer startups that has become pervasive across sectors including high-tech innovative-intensive sectors in the post-2000 period. There has been a secular increase in non-employer entrepreneurship (i.e., self-employment without hiring employees) that has accelerated especially since 2010. The evidence is much of this is secondary or stopgap activity such as being a driver in the fast-growing ridesharing industry.

Accompanying this decline in entrepreneurial activity is an increasing importance in mature and large and even mega firms. This shift in activity towards large, mature firms is of course the flip side of the decline in entrepreneurship. The driving forces of these changes including whether there are changes making entrepreneurship less attractive versus making superstar firms more attractive remain open and active research questions.

While there may be benign factors at work accounting for some of these structural changes, adverse effects are likely at work as well. In the post-2000 period, there has been a decline in young share activity and the increase in mega firms in the high-tech sector. This contrasts with the 1990s when there was a shift towards younger and smaller firms in the high-tech sector. These patterns mimic the rise and fall of aggregate productivity driven by the high-tech sector over this period. Micro evidence also suggests a tight link between innovation, entrepreneurship, and productivity growth in the high-tech sector.

The global pandemic has led to a massive reduction in economic activity and restructuring of daily life. A surprising component of this period of upheaval is a surge in new business applications for likely new employer and non-employer businesses. The surge has been especially large in industries that facilitate remote interactions between businesses and consumers and businesses and workers. It is an open question how much the shift to more remote interactions in the marketplace will persist, but some persistence is likely. The surge in new business applications suggests entrepreneurs will play an important role in this restructuring. Whether this reverses the long-run trend decline in entrepreneurship or is a temporary surge remains to be seen. Even if it is a temporary surge, this highlights the important role that entrepreneurship plays in facilitating structural change in response to changing economic conditions.

## Footnotes

1 Much of the empirical evidence presented in this article reflects the patterns of entrepreneurship in the USA. Evidence from Calvino et al. (2016) shows that many of these patterns hold across OECD countries.

2 The factors underlying the enormous heterogeneity in outcomes for entrepreneurs within narrowly defined sectors is a topic of active research. Managerial ability is likely one of the contributing factors (see, e.g., Lucas, 1978). Recent evidence highlights that the organizational capital created by and embedded in the founding team is important in understanding this heterogeneity (see Choi et al., 2021).

3 I have been fortunate to be part of the research teams developing the BDS and the BFS (see Haltiwanger et al., 2009, and Bayard et al., 2018).

4 The BDS is a public-use database at www.census.gov/ces/dataproducts/bds/index.html.

5 The patterns are quite similar if young is defined as firms five years old or less. An advantage of using the narrower definition is that the patterns can be shown from the early 1980s. In examining those unreported results, the share of activity at young firms peaks around 1987, so starting in 1987, it does little to distort the characterization of the changing age structure of firms.

6 Food and Accommodation Services (NAICS 72) is combined with Retail Trade (NAICS 44–45) for expositional convenience but also because patterns are similar. In the legacy SIC system, much of NAICS 72 was included in Retail Trade.

## Declarations

### Disclaimer

The views expressed in the paper are those of the author alone.
